# l-Lysine-Based Gelators for the Formation of Gels in Water and Alcohol–Water Mixtures

**DOI:** 10.3390/gels9010029

**Published:** 2022-12-30

**Authors:** Yue Miao, Jieying Zhang, Guiju Zhang, Shan He, Baocai Xu

**Affiliations:** School of Light Industry, Beijing Technology and Business University, No. 11 Fucheng Road, Haidian District, Beijing 100048, China

**Keywords:** N^α^, N^ε^-diacyl-l-lysine derivative, alcohol–water mixture, hydrophobic interaction, hydrogen bonding, rheological behavior

## Abstract

N^α^, N^ε^-diacyl-l-lysine and its derivatives are excellent candidates to be used as gelators for the formation of various gels, such as hydrogels, organogels or oleogels, and ionogels. A series of alkali metal salts (Na^+^ and K^+^) of four N^α^, N^ε^-diacyl-l-lysines (acyl including octanoyl, decanoyl, lauroyl and myristoyl) were used to study the gelation behaviors in water and alcohol–water mixtures. l-lysine-based derivatives with long-chain acyl can act as gelators to gel water and alcohol–water mixtures. In contrast, octanoyl and decanoyl derivatives cannot form gels in all solvent systems. Gelation ability, rheological behavior, and morphology vary with the molecular structure of the gelator and the nature of the solvents, as hydrophobic interaction and hydrogen bonding are responsible for the formation of gels. In general, sodium salts performed better in forming gels than their corresponding potassium salts, and myristoyl derivatives were beneficial for gel formation. Although it is challenging to form gels in *t*-butanol–water mixtures, the formed gels show high mechanical strength.

## 1. Introduction

Supramolecular gels are formed by entrapping solvents (i.e., water, oils, organic solvents, ionic liquids, etc.) in a three-dimensional network created by the hierarchical self-assembly of small molecules through weak noncovalent interactions. In recent years, supramolecular gels have gained great interest due to their unique properties and potential applications as a new type of soft material, including applications in cell culture, drug loading and controlled release, antimicrobial therapy, electronic devices, water purification by the removal of heavy metals, and the control of polymorphs by changing the gel network [1,2,3,4].

Various compounds, such as small organic molecules, peptides, nucleobases, saccharides, and coordination complexes, can act as gelators to form gels in water, oils, organic solvents, ionic liquids, etc. [5,6,7,8,9]. Among them, gelators based on amino acids or peptides are of great significance due to their inherent biocompatibility and bioactivities. l-amino acids are widely used as platforms for small molecule gelators, as they are environmentally friendly materials with biocompatibility, biodegradation, and non-toxicity [10,11,12]. In addition, l-amino acids are commercially available at a relatively low price, and their derivatives can be easily prepared by established synthetic methodologies.

Most l-amino acid derivatives are classical amphiphiles that consist of a polar head group (amino acid residues) and one or two hydrophobic tail groups (usually alkyl chains or aromatic groups) [5]. Take l-lysine derivatives as an example. Two amino groups of l-lysine are N-acylated, thus resulting in N^α^, N^ε^-diacyl-l-lysines. Two hydrophobic acyl groups can either be the same, giving symmetrical N^α^, N^ε^-diacyl-l-lysines, or different, giving asymmetrical derivatives. N^α^, N^ε^-diacyl-l-lysine and its derivatives are excellent candidates to be used as gelators for the formation of various gels [13,14,15]. 

Various l-lysine based gelators have been reported recently. N^α^, N^ε^-diacyl-l-lysine with a carboxyl group [16,17] and its ester derivatives [18,19] can form organogels in various organic fluids, such as alkanes, esters, alcohols, ketones, cyclic ethers, polar solvents, aromatic solvents, and mineral and vegetable oils. Long alkyl chains or aromatic rings are the hydrophobic parts that reduce the water solubility of l-lysine gelators. To obtain the relatively better water solubility required to form a hydrogel, one of the simplest strategies is the deprotonation of the carboxylic acid group, which results in a negatively charged carboxylate group. Some of these alkali metal salts (Na^+^ or K^+^ salt) can act as hydrogenators that can gel water, and they also show organogelation abilities for many organic solvents [13,20]. Another approach is to introduce positively charged groups (such as pyridinium salt) into the l-lysine gelator molecules; they act as excellent gelling agents and form hydrogels below 1.0 wt% [21].

So far, it is still challenging to predict gelation properties based on molecular structure alone. Small molecules with similar structures may have very different propensities for gel formation because the solvent is as important as the molecular structure of the gelators in terms of the ability of small molecules to assemble into gels [22]. For amino acid-based gelators, most currently reported studies have focused on gelation of a single solvent: forming hydrogels in water, organogels in organic solvents, oleogels in oils, or ionogels in ionic liquids. The gelation of mixtures of organic solvents and water is challenging and potentially valuable. However, there are only a few reports about the gelation of amino acid gelators in mixed solvents. It is reported that two l-cysteine based gelators can gelate ethanol–water mixtures, thus giving stable and opaque gels [23]. A series of l-alanine-derived amphiphilic molecules were observed to form stable organogels in organic solvents in the presence of a small amount of water, whereas no gelation was found in pure organic solvents [24].

**Figure 1:**
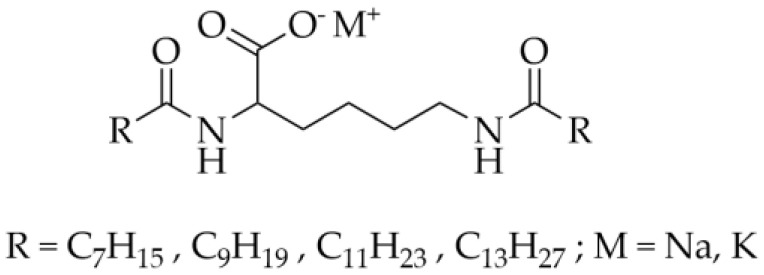
The molecular structure of N^α^, N^ε^-diacyl-l-lysine derivatives studied in the current study.

Based on our previous study about the gelation properties of four N^α^, N^ε^-diacyl-l-lysines in vegetable oils [25], the corresponding alkali metal salts (Figure 1) were obtained by converting the carboxylic acids to carboxylate salts in this study. The sodium and potassium salts of N^α^, N^ε^-diacyl-l-lysines (i.e., N^α^, N^ε^-dioctanoyl-l-lysine; N^α^, N^ε^-didecanoyl-l-lysine; N^α^, N^ε^-dilauroyl-l-lysine; N^α^, N^ε^-dimyristoyl-l-lysine) are referred to as 2C8-Lys-Na, 2C10-Lys-Na, 2C12-Lys-Na, 2C14-Lys-Na, 2C8-Lys-K, 2C10-Lys-K, 2C12-Lys-K, and 2C14-Lys-K. The gelation behavior of these alkali metal salts in water as well as in organic solvent–water mixtures was explored. The driving forces for gelation in water as well as the rheological properties and morphology of the formed gels were also studied.

## 2. Results and Discussion

### 2.1. Gelation of Water

**Figure 2:**
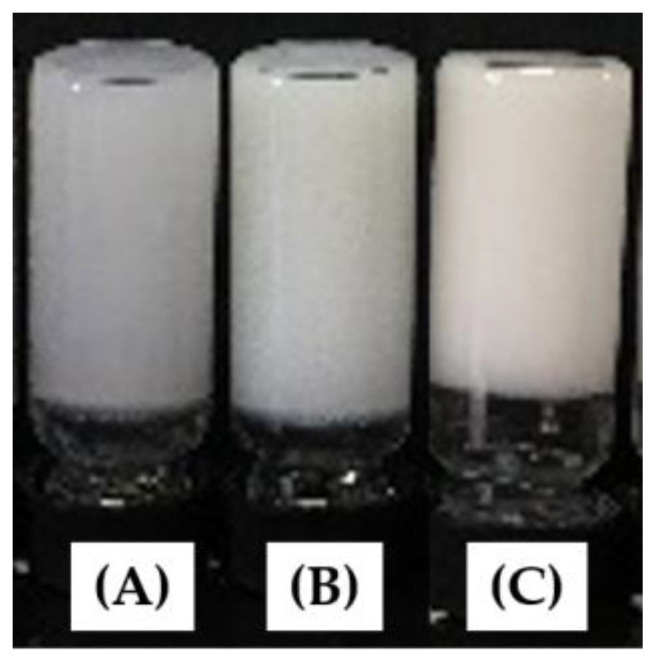
Photographs of vials showing gelation of gelators in water: (**A**) 2C12-Lys-Na, (**B**) 2C12-Lys-K, and (**C**) 2C14-Lys-K.

For all of the sodium and potassium salt of N^α^, N^ε^-diacyl-l-lysines, only 2C12-Lys-Na, 2C12-Lys-K, and 2C14-Lys-K functioned as hydrogelators that could gel water at a minimum gelation concentration (MGC) of 1 mg/mL, 5 mg/mL, and 2.3 mg/mL, respectively, within 10 min (Figure 2). Compounds with shorter carbon chain lengths, i.e., 2C8-Lys-Na, 2C10-Lys-Na, 2C8-Lys-K, and 2C10-Lys-K, produced viscous aqueous solutions above 1 mg/mL. However, 2C14-Lys-Na was found to be poorly soluble even when heated at 80 °C, which is probably due to its long hydrophobic chains and sodium salt.

### 2.2. Gelation of Alcohol–Water Mixtures

**Figure 3:**
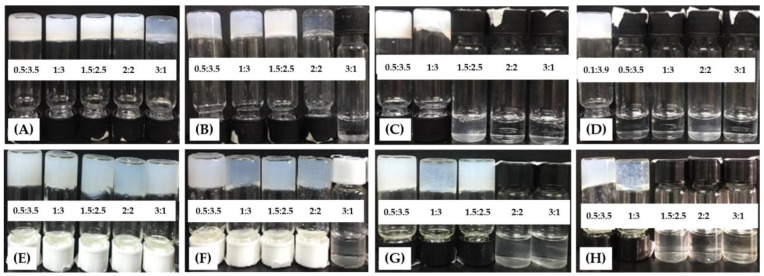
Photographs of vials showing gelation of gelators in alcohol–water mixtures with different volume ratios (*v*/*v*). (**A**–**D**): 2C12-Lys-Na in methanol–H_2_O, ethanol–H_2_O, isopropanol–H_2_O, and *t*-butanol–H_2_O; (**E**–**H**): 2C14-Lys-Na in methanol–H_2_O, ethanol–H_2_O, isopropanol–H_2_O, and *t*-butanol–H_2_O.

Four short-chain alcohols, i.e., methanol, ethanol, isopropanol, and *t*-butanol, were used as organic solvents for the gelation behavior study of the sodium and potassium salts of N^α^, N^ε^-diacyl-l-lysines. None of the eight compounds could form organogels in pure organic solvents. In the alcohol–water mixtures, 2C12-Lys-Na, 2C12-Lys-K, 2C14-Lys-Na, and 2C14-Lys-K formed translucent-to-white and thermoreversible gels, whereas compounds with shorter carbon chain lengths did not. Figure 3 displays the photographs of vials showing gelation of 2C12-Lys-Na and 2C14-Lys-Na in alcohol–water mixtures with different volume ratios (*v*/*v*). Moreover, the gelation behaviors of 2C12-Lys-Na, 2C12-Lys-K, 2C14-Lys-Na, and 2C14-Lys-K have been summarized in Table 1.

It can be seen from Table 1 that 2C12-lys-Na and 2C14-lys-Na formed gels in methanol–water mixtures with different volume ratios. The smallest MGC was obtained for 2C12-lys-Na and 2C14-lys-Na when the methanol–water volume ratio was 1.5:2.5. When gelation occurred in ethanol–water mixtures, 2C12-lys-Na and 2C14-lys-Na formed gels except for the ethanol–water volume ratio of 3:1, and the smallest MGC was also obtained at 1.5:2.5. It was harder to form gels for 2C12-lys-Na and 2C14-lys-Na in isopropanol–water and *t*-butanol–water mixtures, especially for 2C12-lys-Na in *t*-butanol–water mixtures; gels only formed when the *t*-butanol–water volume ratio was at 0.1:3.9, with an MGC of 22.5 mg/mL.

As for potassium salts, 2C12-Lys-K exhibited poor gelation ability in all alcohol–water mixtures. In contrast, 2C14-Lys-K tended to function as a more effective gelator. Similarly, the gelation ability of the same gelator decreased with the increased carbon chain lengths of alcohols. In general, the sodium salts performed better in forming gels than their corresponding potassium salts. This result is inconsistent with those reported in the literature [20], which may be due to the differences in the molecular structure and solvents.

The speed of gel formation in the alcohol–water mixtures was slower than in water, although gels formed within 30 min in all cases. In general, the gel formation was faster with a lower proportion of alcohol and lower MGC, thus resulting in shorter gelation time. However, there was little difference in gel time for different types of alcohols.

### 2.3. Driving Forces for Gelation in Water

The hydrophobic interaction and hydrogen bonding were the main driving forces for the self-assembly of the amino acid-based gelators. Fluorescence and FT-IR study were carried out to evaluate the driving forces of the gel formation.

#### 2.3.1. Hydrophobic Interaction

To elucidate the self-assembly process of a gelator, 8-anilino-1-naphthalenesulfonic acid (ANS) was used as a probe to study its luminescence spectra [21]. ANS is a commonly used fluorescence probe in hydrophobic environments. The fluorescence of ANS significantly depends on the polarity of its environment; the very weak ANS fluorescence in water significantly increases and blue-shifts to shorter wavelengths in hydrophobic environments [20].

**Figure 4:**
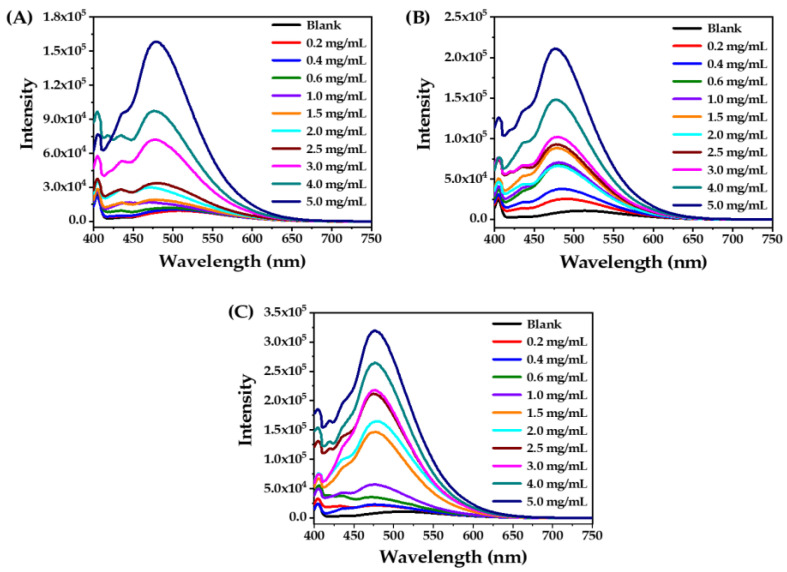
Luminescence spectra of ANS (1.0 × 10^−5^ mol/L) in aqueous solutions containing various concentrations of 2C8-Lys-Na (**A**), 2C10-Lys-Na (**B**), and 2C12-Lys-Na (**C**).

**Figure 5:**
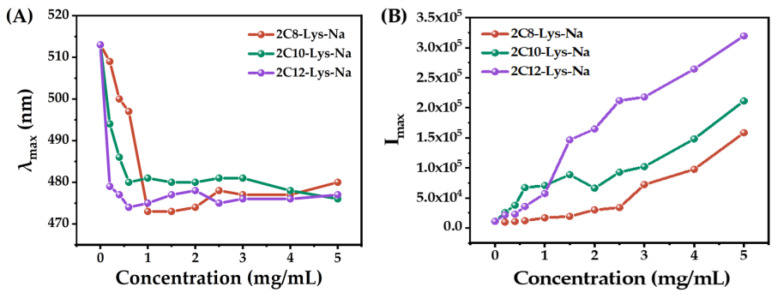
Dependence of (**A**): luminescence maxima (λ_max_) and (**B**): maximum luminescence intensities (*I*_max_) on the concentration of 2C8-Lys-Na, 2C10-Lys-Na, and 2C12-Lys-Na.

The fluorescence spectra of ANS in aqueous solutions at various concentrations of 2C8-Lys-Na, 2C10-Lys-Na, and 2C12-Lys-Na are shown in Figure 4. Unfortunately, poor water solubility prevented the investigation of 2C14-Lys-Na. Figure 5 shows the dependence of luminescence maxima (λ_max_) and maximum luminescence intensities (*I*_max_) on the concentration of 2C8-Lys-Na, 2C10-Lys-Na, and 2C12-Lys-Na.

In up to 1 mg/mL of 2C8-Lys-Na, the λ_max_ blue-shifted from 513 to 473 nm with increasing concentration (Figure 5A). Further addition of 2C8-Lys-Na increased the luminescence intensity, but the value of λ_max_ did not change much. The luminescent behavior indicates that the ANS molecules are embedded into a hydrophobic environment, i.e., the interior of the self-assembled aggregates is hydrophobic. For 2C10-Lys-Na and 2C12-Lys-Na, the critical concentrations were 0.6 mg/mL and 0.2 mg/mL, respectively. This indicates that 2C12-Lys-Na blue-shifts faster to short wavelengths. In addition, the extent of change in *I*_max_ with increasing concentration is also different among the three compounds (Figure 5B). Compared to 2C8-Lys-Na and 2C10-Lys-Na, 2C12-Lys-Na exhibits a faster change of *I*_max_, especially after surpassing the concentration of 1 mg/mL, which is consistent with its MGC.

The above results illustrate that the aqueous solutions of the three compounds showed hydrophobic interaction, but there are differences due to the different carbon chain lengths. The salt 2C12-Lys-Na, with a longer carbon chain length, appeared to have stronger hydrophobic interactions in the self-assembly process. Correspondingly, 2C12-Lys-Na acted as a gelator in the water giving a stable hydrogel, whereas 2C8-Lys-Na and 2C10-Lys-Na did not. Therefore, this implies that hydrophobic interaction is an important driving force for hydrogel formation.

#### 2.3.2. Hydrogen Bonding

The hydrogen bonding for the gel formation process was carried out by an FT-IR study. Table 2 shows the FT-IR data of 2C12-Lys-Na, 2C14-Lys-Na, and dried gels formed in this study. For the gelators of 2C12-Lys-Na and 2C14-Lys-Na, the typical amide bonds in the absorption bands of the N–H stretching vibration (about 3327 cm^−1^), amide I (about 1641 cm^−1^) and amide II (about 1540 cm^−1^), were observed. When 2C12-Lys-Na formed into a hydrogel in water, the absorption bands were observed at 3326 cm^−1^ for the N–H stretching vibration, 1642 cm^−1^ for amide I, and 1555 cm^−1^ for amide II. This blue-shift of amide II indicates the presence of intermolecular hydrogen bonds of amide groups [20]. The amide II of the gels formed in organic solvent–water mixtures also blue-shifted to the range of 1551–1560 cm^−1^. In addition, the absorption bands of the N–H stretching vibration red-shifted to a lower frequency. Compared to the gels formed by 2C12-Lys-Na, greater red-shifts of N–H stretching vibration absorption bands were displayed for the gels formed by 2C14-Lys-Na, which means stronger hydrogen bonding interactions occurred. This phenomenon is compatible with the gelation ability of 2C12-Lys-Na and 2C14-Lys-Na in organic solvent–water mixtures. The FT-IR results suggest that hydrogen bonding is also one of the driving forces for self-assembly into nanostructures followed by gelation.

### 2.4. Rheological Properties

**Figure 6:**
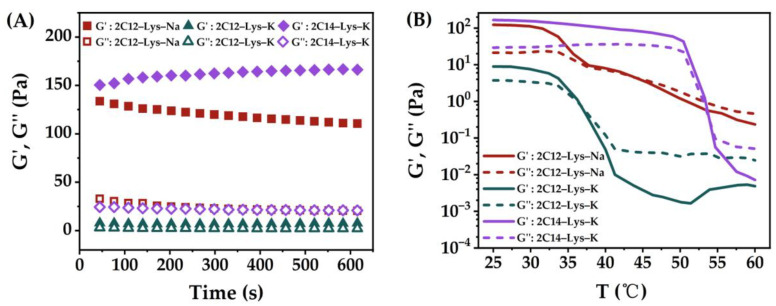
Rheological behaviors of gels formed by 2C12-Lys-Na in water: (**A**) Variation of storage modulus (G’) and loss modulus (G″) with time; (**B**) dependence of the storage modulus (G’) and loss modulus (G″) with the temperature.

Rheological properties are an important factor affecting the potential applications of the gels. To characterize the general rheological behavior and to provide information on the stiffness of aggregation, the hydrogels of 2C12-Lys-Na, 2C12-Lys-K, and 2C14-Lys-K in water at room temperature have been examined. The storage modulus (G’) and loss modulus (G″) results are shown in Figure 6A. In all cases, the storage modulus (G’) was larger than the loss modulus (G″), and the storage modulus G’ was mainly time-independent, thus indicating the formation of a stable hydrogel. In general, the higher the storage modulus (G’), the stronger the corresponding gel strength. The results show that 2C12-Lys-Na and 2C14-Lys-K formed stronger hydrogels in water than 2C12-Lys-K. This conclusion can also be drawn from the temperature dependence of G’ and G″ (Figure 6B). The phase transition temperatures of 2C12-Lys-Na, 2C12-Lys-K, and 2C14-Lys-K hydrogels were 43.5 °C, 36.8 °C, and 54.0 °C, as listed in Table 3.

**Figure 7:**
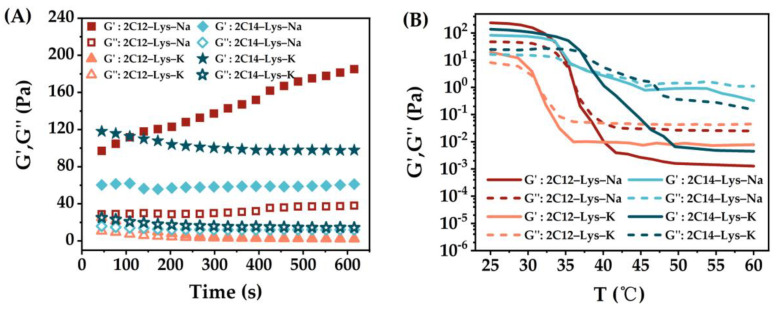
Rheological behaviors of gels formed by 2C12-Lys-Na, 2C12-Lys-K, 2C14-Lys-Na, and 2C14-Lys-K in methanol–water mixtures: (**A**) Variation of storage modulus (G’) and loss modulus (G″) with time; (**B**) dependence of the storage modulus (G’) and loss modulus (G″) with the temperature.

**Figure 8:**
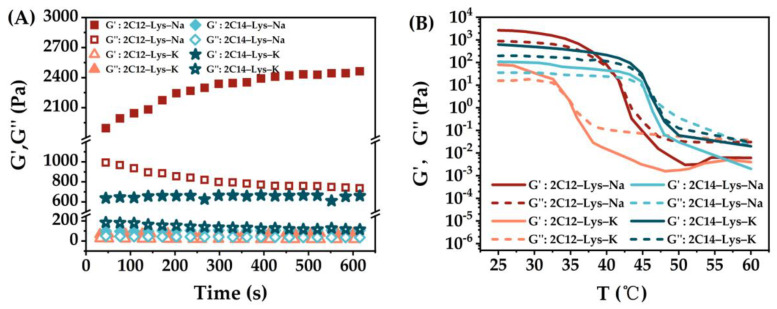
Rheological behaviors of gels formed by 2C12-Lys-Na, 2C12-Lys-K, 2C14-Lys-Na, and 2C14-Lys-K in ethanol–water mixtures: (**A**) Variation of storage modulus (G’) and loss modulus (G″) with time; (**B**) dependence of the storage modulus (G’) and loss modulus (G″) with the temperature.

**Figure 9:**
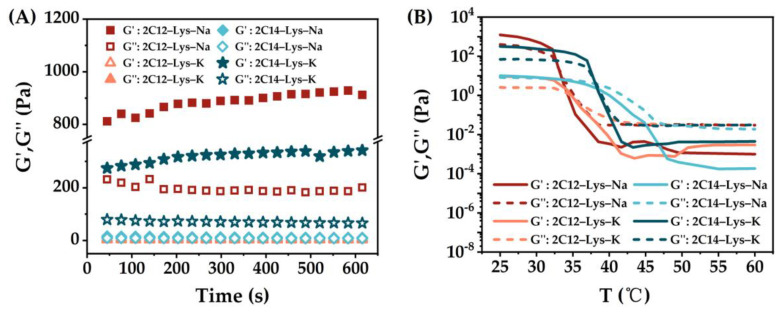
Rheological behaviors of gels formed by 2C12-Lys-Na, 2C12-Lys-K, 2C14-Lys-Na, and 2C14-Lys-K in isopropanol–water mixtures: (**A**) Variation of storage modulus (G’) and loss modulus (G″) with time; (**B**) dependence of the storage modulus (G’) and loss modulus (G″) with the temperature.

**Figure 10:**
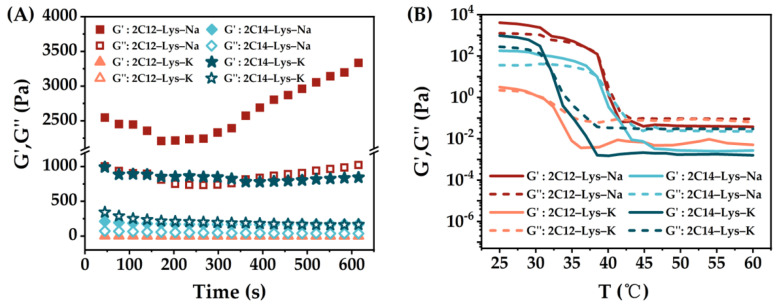
Rheological behaviors of gels formed by 2C12-Lys-Na, 2C12-Lys-K, 2C14-Lys-Na and 2C14-Lys-K in *t*-butanol–water mixtures: (**A**) Variation of storage modulus (G’) and loss modulus (G″) with time; (**B**) dependence of the storage modulus (G’) and loss modulus (G″) with the temperature.

The rheological behaviors of gels formed by 2C12-Lys-Na, 2C12-Lys-K, 2C14-Lys-Na, and 2C14-Lys-K in methanol–water, ethanol–water, isopropanol–water and *t*-butanol–H_2_O mixtures were also investigated, and the results are shown in Figure 7, Figure 8, Figure 9 and Figure 10. The phase transition temperature of each gel is summarized in Table 3. Similarly, the storage modulus (G’) is higher than the loss modulus (G″) for all samples, confirming the formation of stable gels. 

When 2C12-Lys-Na was used as a gelator, the gels formed in *t*-butanol–water and ethanol–water mixtures exhibited obviously high viscoelasticity, indicating the higher mechanical strength of gels. In comparison, the gels formed in methanol–water mixtures show low viscoelasticity. Similar behaviors are observed for the other three gelators. These results are not consistent with the gelation ability of each gelator in different alcohol–water mixtures. Although it was difficult to form gels in *t*-butanol–water mixtures, the formed gels show high mechanical strength. On the contrary, the gel formation was relatively easy in methanol–water mixtures, giving low mechanical strength gels. When gelating in the same alcohol–water mixtures, gels formed by 2C12-Lys-Na showed relatively high viscoelasticity, followed by 2C14-Lys-K, and then 2C14-Lys-Na. At the same time, the gels formed by 2C12-Lys-K displayed the lowest viscoelasticity with weak gel strength.

It can be seen from Figure 7B, Figure 8B, Figure 9B, and Figure 10B that the rheological behaviors of all gels formed in this study are temperature-dependent. For each gel, the values of G’ and G″ gradually decreased as the temperature increased. G’ was higher than G″ at the beginning, indicating that solid behavior dominated. After a specific temperature, G″ surpassed G’, indicating liquid behavior now dominated. The temperature at this phase transition point can be defined as the phase transition temperature of a gel. Among the four gelators, gels formed by 2C14-Lys-K showed better thermal stability except in the *t*-butanol–water mixtures, while gels formed by 2C12-Lys-K displayed relatively weak thermal stability except in the isopropanol–water mixtures.

### 2.5. Morphology of Gels

**Figure 11:**
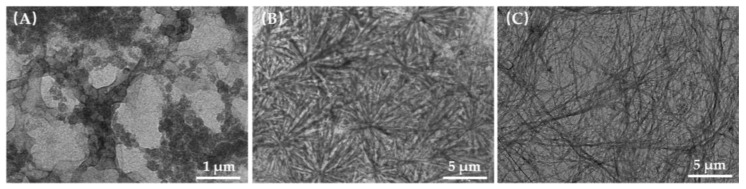
TEM images of self-assembled aggregates in water for 2C8-Lys-Na (**A**)**,** 2C10-Lys-Na (**B**), and dried hydrogel formed by 2C12-Lys-Na (**C**).

Gelator molecules have been demonstrated to self-assemble into structurally defined nanoscale superstructures, including nanofibers, nanotubes, and nanosheets in a supramolecular gel. As discussed before, 2C12-Lys-Na can form hydrogels in water instead of 2C8-Lys-Na and 2C10-Lys-Na. To obtain visual insights into the self-aggregation mode of l-lysine based gelators in aqueous solution, TEM images of self-assembly aggregates for 2C8-Lys-Na and 2C10-Lys-Na, as well as the dried hydrogel formed by 2C12-Lys-Na in water, were produced as shown in Figure 11. Macroscopically, the aqueous solution of 2C8-Lys-Na (5 mg/mL) is transparent with good fluidity. From Figure 11A, a spherical shape of self-assembled aggregates can be observed with a size of about 200 nm. The aqueous solution of 2C10-Lys-Na is also transparent with high viscosity and poor fluidity, but no gel formation. Massive spherulite-like aggregates were observed in Figure 11B. As for 2C12-Lys-Na, it can be seen from Figure 11C that the gelator molecules self-assemble into fibrous structures through noncovalent interactions, and then the fine fibers are entangled to form a network. This hierarchical network structure is mainly responsible for the gelation of 2C12-Lys-Na. These results are consistent with the gelation behaviors of the three compounds.

**Figure 12:**
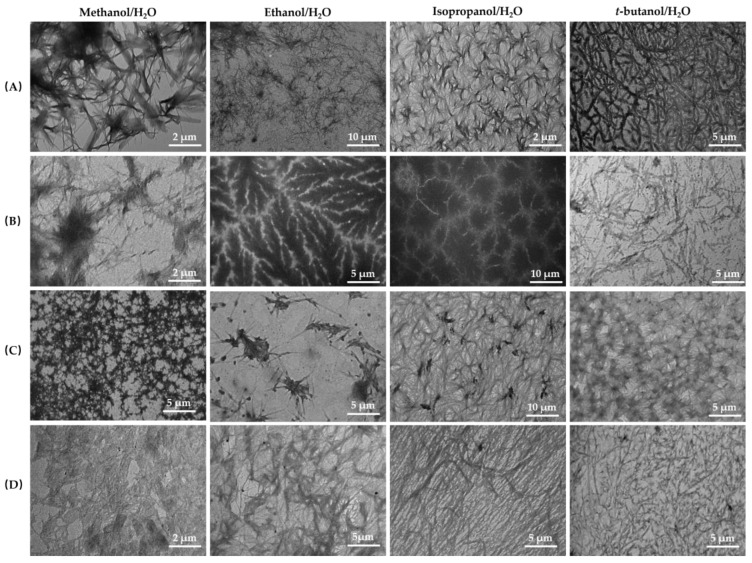
TEM images of dried gels formed in alcohol–water mixtures by (**A**) 2C12-Lys-Na, (**B**) 2C14-Lys-Na, (**C**) 2C12-Lys-K, and (**D**) 2C14-Lys-K.

To explore their gelation behavior and microscopic morphology, TEM images of dried gels formed in alcohol–water mixtures by 2C12-Lys-Na, 2C14-Lys-Na, 2C12-Lys-K, and 2C14-Lys-K were taken and shown in Figure 12. It is well-established that gelation behavior and microscopic morphology depend on the molecular structure as well as on the solvents. It can be seen from Figure 12 that the morphology of gels varies in different alcohol–water mixtures when formed by the same gelators. In the same alcohol–water mixtures, different morphologies of gels formed by different gelators were also observed. In most cases, these gelators formed a supramolecular nanofiber, and their entanglements created a three-dimensional network. Therefore, gelation occurs by immobilizing solvents in nanospaces in the three-dimensional network. Combined with their rheological behavior, gels with longer and more entangled fibers exhibit higher viscoelasticity with strong gel strength, such as the gel formed by 2C12-Lys-Na in *t*-butanol–water mixtures. As for gels formed by 2C12-Lys-K in the alcohol–water mixtures, short needle-like fibers and their small cluster aggregates were observed, which may lead to their lower viscoelasticity with weak gel strength. Gel strength mainly depends on the compactness of the three-dimensional network in the gels [14]. Short ribbon-like aggregates caused less entanglement and, hence, viscoelasticity [24].

## 3. Conclusions

The sodium and potassium salts derived from four N^α^, N^ε^-diacyl-l-lysines were used to study the gelation behaviors in water and alcohol–water mixtures. 2C12-lys-Na, 2C12-lys-K, and 2C14-lys-K formed hydrogels in water. The salts 2C12-lys-Na, 2C14-lys-Na, 2C12-lys-K, and 2C14-lys-K formed gels in four alcohol–water mixtures. Gelation ability is not only related to the molecular structure of the gelators, but it is also affected by the solvents. The rheological properties and morphology of the formed gels were also affected by both gelators and solvents, as hydrophobic interaction and hydrogen bonding were the main driving forces for the gel formation. In most cases, gelator molecules self-assembled in the solution into supramolecular nanofibers, and their entanglements yielded a three-dimensional networkthat could entrap solvents, thus resulting in gelation. In this paper, interesting gelation behaviors of l-lysine-based gelators was found in alcohol–water mixtures. However, the gelation mechanism, such as the specific gelation process, the role of organic solvents, and the intermolecular interaction, all remain unclear, and in-depth research will be conducted in a further study.

## 4. Materials and Methods

### 4.1. Materials

8-anilino-1-naphthalenesulfonic acid (ANS) was obtained from Shanghai Aladdin Biochemical Technology Co., Ltd (Shanghai, China). Methanol, ethanol, isopropanol, and *t*-butanol were purchased from Shanghai Macklin Biochemical Technology Co., Ltd. (Shanghai, China). Four N^α^, N^ε^-diacyl-l-lysines (acyl including octanoyl, decanoyl, lauroyl and myristoyl) were synthesized using the previous method [26]. The alkali metal salts (Na^+^ and K^+^) of N^α^, N^ε^-diacyl-l-lysines were simply achieved by conversion of carboxylic acid to a carboxylate by neutralizing with sodium hydroxide and potassium hydroxide.

### 4.2. Gelation Test

The preparation of gels was conducted according to the method reported in the literature [26]. A known amount of the potential gelator and 4 mL solvent (water or alcohol–water mixtures) were placed into a screw-cap vial and heated (approximately 80 °C) until the gelator dissolved completely. The resulting solution was naturally cooled to room temperature. Gel formation was estimated using the vial inversion method.

### 4.3. FT-IR Study

FT-IR spectra were determined on a Nicolet iS10 FT-IR Spectrometer (Thermo Fisher Scien-tific, Madison, WI, USA) using KBr tablets at room temperature [27]. Wet gels were freeze-dried and crushed into powder, and then mixed with KBr powder and pressed into tablets.

### 4.4. Fluorescence Study

Fluorescence spectra were investigated using an FLS1000 fluorescence spectrometer (Edinburgh Instrument, Livingston, UK). A series of aqueous solutions of three water-soluble gelators, i.e., 2C8-Lys-Na, 2C10-Lys-Na, and 2C12-Lys-Na, were prepared with different concentrations (in the range of 0–5 mg/mL). The concentration of ANS was 1.0 × 10^−5^ mol/L. The samples were measured with the excitation wavelength at 356 nm, which corresponds to the absorption maximum [21].

### 4.5. Rheological Behavior Measurements

The rheological properties of the gels were measured using a HAAKE MARS III rheometer (Thermo Electron GmbH, Dreieich, Germany) according to the reported method with slight modification [28]. The storage modulus (G’) and loss modulus (G″) over time were measured at 25 °C with a constant rotational speed of 1 rad/s and a shear strain of 0.25 Pa. The storage modulus (G’) and loss modulus (G″) were also measured at a heating temperature from 25 °C to 60 °C, with a fixed rotational speed of 1 rad/s and a shear strain of 0.25 Pa.

### 4.6. TEM Measurements

Gel morphology was observed with a transmission electron microscope. The samples were prepared according to the method in the literature [7,25]. First, a small amount of gel was cast on a carbon-coated copper grid (300 mesh) and allowed to air dry at room temperature before observation. The microscopic images of the gels were monitored using an X-MAX JEM-2100 transmission electron microscope (JEOL, Tokyo, Japan) operating at 120 kV.

## Figures and Tables

**Table 1:** Gelation behaviors of 2C12-Lys-Na, 2C12-Lys-K, 2C14-Lys-Na, and 2C14-Lys-K in alcohol–water mixtures with different volume ratios (*v*/*v*).

Solvents	Volume Ratio (*v*/*v*)	2C12-Lys-Na	2C14-Lys-Na	2C12-Lys-K	2C14-Lys-K
methanol–H_2_O	3:1	46 (23)	56 (26)	S	33.9 (29)
	2:2	8 (19)	8.2 (24)	S	13.1 (27)
	1.5:2.5	3.5 (17)	6.4 (20)	57.8 (25)	10.3 (26)
	1:3	5 (18)	7.9 (21)	24.5 (26)	15.4 (25)
	0.5:3.5	7.5 (19)	18.6 (21)	17.5 (23)	35 (25)
ethanol–H_2_O	3:1	S	S	S	22 (26)
	2:2	24.5 (20)	4.4 (24)	S	14 (23)
	1.5:2.5	2 (17)	2 (19)	S	5.6 (22)
	1:3	5 (18)	9 (20)	5	7.8 (21)
	0.5:3.5	6 (18)	22.4 (20)	3.6	13.1 (21)
isopropanol–H_2_O	3:1	S	S	S	S
	2:2	S	S	S	S
	1.5:2.5	S	16.4 (21)	S	33.4 (26)
	1:3	29.5 (22)	15.2 (21)	S	51.7 (26)
	0.5:3.5	11 (19)	8 (20)	31	15 (25)
*t*-butanol–H_2_O	3:1	S	S	S	S
	2:2	S	S	S	S
	1.5:2.5	S	S	S	S
	1:3	S	8.8 (22)	S	S
	0.5:3.5	S	13 (23)	S	34.6 (26)
	0.1:3.9	22.5 (23)	12.3 (21)	33.1	18.1 (23)

Notes: Values denote minimum gel concentration (MGC, mg/mL); S denotes solution; data in parentheses indicate gelation time (min).

**Table 2:** FT-IR data (cm^−1^) of 2C12-Lys-Na, 2C14-Lys-Na, and dried gels formed in this study.

Compounds	Solvents	N–H Stretching Vibration	Amide I	Amide II
2C12-Lys-Na	powder ^1^	3327	1641	1540
	H_2_O	3326	1642	1555
	Methanol–H_2_O	3318	1642	1557
	Ethanol–H_2_O	3309	1643	1556
	Isopropanol–H_2_O	3314	1642	1552
	*t*-butanol–H_2_O	3316	1640	1548
2C14-Lys-Na	powder ^1^	3328	1640	1542
	Methanol–H_2_O	3309	1639	1558
	Ethanol–H_2_O	3306	1640	1560
	Isopropanol–H_2_O	3308	1642	1553
	*t*-butanol–H_2_O	3301	1639	1558

^1^ Powder of gelators.

**Table 3:** The phase transition temperature of gels formed by l-lysine-based gelators.

Solvents	Phase Transition Temperature (°C)			
	2C12-Lys-Na	2C14-Lys-Na	2C12-Lys-K	2C14-Lys-K
H_2_O	43.5	-	36.8	54.0
methanol–H_2_O	35.9	36.7	31.2	37.0
ethanol–H_2_O	41.1	45.4	34.4	45.9
isopropanol–H_2_O	33.1	32.4	34.9	38.2
*t*-butanol–H_2_O	37.7	38.5	31.0	32.0
